# *QuickStats:* Age-Adjusted Death Rates* for Motor Vehicle Traffic Injury^^†^^ — United States, 2019

**DOI:** 10.15585/mmwr.mm7011a4

**Published:** 2021-03-19

**Figure:**
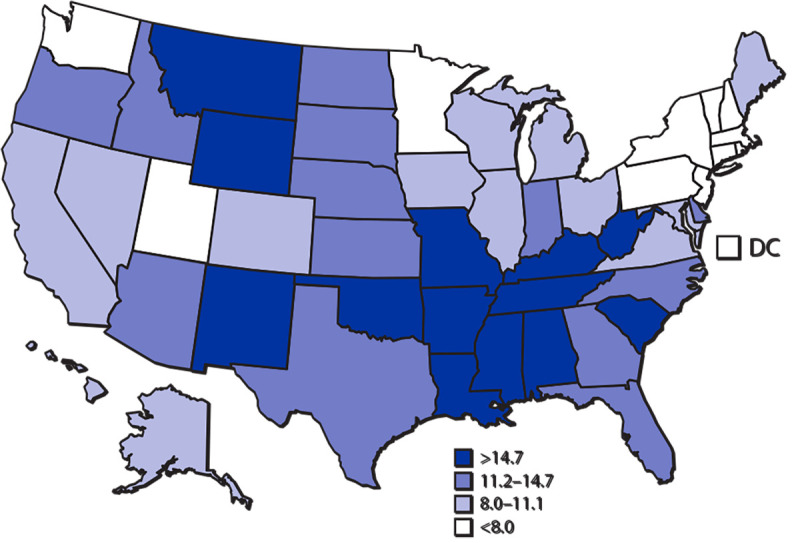
In 2019, the death rate in the United States for motor vehicle traffic injury was 11.1 per 100,000 standard population. The four states with the highest age-adjusted death rates were Mississippi (24.2), Alabama (19.8), New Mexico (19.1), and South Carolina (18.9). The four jurisdictions with the lowest age-adjusted death rates were Rhode Island (6.1), District of Columbia (6.1), New York (5.1), and Massachusetts (4.9).

**Source:** National Vital Statistics System, Mortality, 2019. https://www.cdc.gov/nchs/nvss/deaths.htm

For more information on this topic, CDC recommends the following link: https://www.cdc.gov/transportationsafety

